# Blood loss and prolonged air leak reduction by applying TenaTac^®^ gelatine patch after major pulmonary minimal-invasive resection

**DOI:** 10.1007/s11748-025-02194-3

**Published:** 2025-09-10

**Authors:** Caroline Rivera, Cyril Perrot, Florence Mazeres, Elodie Rive

**Affiliations:** 1 Thoracic Surgery Department, Centre Hospitalier de la Cote Basque, 13, avenue de l’Interne Jacques Loeb, 64100 Bayonne, France; 2 Medical Advices Consulting, Lëtzebuerg, Luxembourg

**Keywords:** Major lung resection, Sealant, Hemostasis, Aerostasis, Prolonged air leak

## Abstract

**Objective:**

Reduction of bleeding and prolonged air leak (>5 days) following major lung resection remains a challenge. Hemostasis and aerostasis devices can facilitate earlier pleural de-drainage and fast-track. Our objectives were to evaluate the efficacy of TenaTac^®^ (an elastic, adhering patch approved as a medical device) in reducing bleeding and prolonged air leak after major lung resection.

**Methods:**

This monocentric retrospective case–control study, using prospectively collected data, includes 60 patients who underwent, between 2022 and 2024, minimally invasive robot-assisted lobectomy or segmentectomy: 30 with TenaTac^®^ *vs.* 30 with other devices. Data were extracted from Epithor, the French national database, with ethics committee validation.

**Results:**

Patients characteristics, Index of Prolonged Air Leak, and surgical procedures were similar in both groups (NS). TenaTac^®^ hemostatic benefit was comparable to other devices (*p* = 0.56). Prolonged air leak rate was significantly lower with TenaTac^®^ (3%) than for control devices (37%) (*p* = 0.0004). Post-operative air leakage duration was significantly shorter in TenaTac^®^ group than in control group (2.23 ± 2.57 *vs.* 4.23 ± 3.87 days, *p* = 0.01). Mean drainage duration and length of stay were both reduced with TenaTac^®^ by 36 hours. No significant difference was observed between the two groups in terms of morbidity (90-day post-operative complications classified as Clavien–Dindo grade>II, *p* = 0.33), readmission rates (nil), or 90-day mortality (no deaths).

**Conclusions:**

Numerous hemostatic or aerostatic devices have been previously evaluated without achieving consensus in the prevention of prolonged air leak. As an absorbable, adherent gelatine patch, TenaTac^®^ significantly reduces the incidence of prolonged air leak after major lung resection.

## Introduction

Post-operative complications following major lung resection are the primary factors hindering the early discharge of thoracic surgery patients. Their incidence ranges from 20% to 30% [1], irrespective of the underlying indication, surgical technique, or approach. As is well documented, patients undergoing treatment with anticoagulants or antiplatelet therapy, or those afflicted with a bleeding disorder, are predisposed to perioperative and post-operative bleeding [2]. In the event of post-operative bleeding or prolonged air leak (PAL), the removal of the chest tube is postponed and may be associated with a prolonged hospital stay or readmission, resulting in higher healthcare costs.

Intraoperative control of bleeding and air leakage includes careful dissection, cauterization, suturing techniques, stapling [3–5], and the use of sealants may also be suggested [6–9].The repair of parenchymal defects, whether due to obligatory fissure dissection or accidental lung injuries associated with adhesions or manipulation of the lobes, can be challenging depending on the fragility of the lung and the elasticity of the lung tissue. In practical terms, PAL has been identified as the main limitation in enhanced recovery after surgery (ERAS) [10, 11], also referred to as *fast-track*. The utilization of predictive scores [12–14] has enabled surgeons to preoperatively identify patients who are at increased risk of PAL, thus facilitating the anticipation of additional preventive operative procedures, such as the use of sealants. It has been demonstrated that the use of prophylactic sealants at the time of pulmonary resection leads to a substantial reduction in both the volume and frequency of blood loss and PAL. Consequently, the duration required for drain removal, as well as the overall length and expense of hospitalization, is significantly decreased without compromising clinical outcomes or worsening the post-operative course (in terms of morbidity, complications, serious adverse events or mortality) [8]. However, the high manufacturing costs of certain sealants and the lack of statistically significant benefit highlight the need to rationalize their use [15, 16].

In recent years, there has been considerable interest in the potential of gelatine-based medical devices for sealing lung defects [17]. A further advantage of these products is their low cost. However, it should be noted that these devices all contain an additive to enable their adhesive functionality. In this context, decision was taken to evaluate TenaTac^®^, a gelatine patch with a physical surface modification that achieves adhesion, without the use of additives [18]. As TenaTac^®^ was launched in the French market, we employed it to achieve optimal hemostasis in instances of minor bleeding such as oozing from fissures or lymph nodes dissection zones or from stapler lines. We observed TenaTac^®^’s considerable adhesion and elastic properties resulting in the sealing of air leaks concurrently on the same zones.

The primary objective of this study is to evaluate the efficacy of TenaTac^®^ in reducing blood loss following major lung resection in comparison with sealing devices commonly used by thoracic surgeons for the same indication. The secondary objective is to evaluate its efficacy in reducing PAL in these patients.

## Subjects

We set up a single-center case–control study involving 60 patients who underwent robot-assisted lobectomy or segmentectomy in our department of Thoracic Surgery at the Centre Hospitalier de la Côte Basque (Bayonne, France), between February and December 2022 and then between August 2023 and April 2024 for the control group.

30 patients in the control group underwent surgery consecutively and were treated with the sealing agents that were available at Bayonne Hospital at the time. The patients in the experimental group were also operated on consecutively, for the same indications, under the same conditions and following the same procedures as those in the control group. However, these operations took place after the TenaTac^®^ patch had been launched in the French market and a short period devoted to testing this innovation. Furthermore, a period of waiting was required for the patch to be referenced by the hospital and utilized on a routine basis. That is why there are no patients included between January and July 2023.

### Patients and surgical procedures

The experimental group (EG) comprised 30 patients. These patients, who underwent surgery between August 2023 and April 2024, were treated with TenaTac^®^ to achieve hemostasis and seal parenchymal defects resulting from surgical dissection. The remaining 30 patients, constituting the control group (CG), underwent surgery between February and December 2022 and received alternative sealants under comparable circumstances and for analogous indications.

The baseline demographics comprised a range of variables, including age, gender, body mass index (BMI), American Society of Anesthesiologists (ASA) score, World Health Organization performance status (PS), forced expiratory volume (FEV), dyspnea score according to Medical Research Council, Index of Prolonged Air Leak (IPAL) [12, 13], and number of comorbid diseases. The number of comorbidities per patient, considered as a categorical variable, was taken into account in the descriptive analyses on the basis of literature data suggesting the superiority of this variable in predicting in-hospital mortality [19].

The following variables were also collected for each surgical procedure: type (segmentectomy or lobectomy), localization (right upper lobe, RUL; middle lobe, ML; right lower lobe, RLL; left upper lobe, LUL; left lower lobe, LLL), surgery duration, and pre-operative hemoglobin level (g/dl). All patients underwent robotic surgical procedure using the Da Vinci^®^ X robot and received one or other of the sealants available at the hospital during the specified time period. The surgical procedures were performed by three thoracic surgeons, who all employed the same standardized technique of robot-assisted thoracic surgery (RATS). The robotic technique employed within our institution has been standardized since 2015. We strictly respect the so-called “French lobectomy” technique [20]. All three thoracic surgeons within the department have received training in this technique. Furthermore, 3D pre-operative reconstructions are systematically employed in our daily practice, encompassing both lobectomies and segmentectomies. In our practice, various techniques are employed to control air leaks. The primary objective is prevention; therefore, the attempt is made to dissect as little parenchyma as possible in the fissure in order to avoid the occurrence of an air leak. Second, the use of staplers to seal fissures is employed to mitigate parenchymal air leak, in conjunction with direct suturing of defects. However, this approach is frequently insufficient, particularly in patients with emphysema or weakened parenchyma due to neo-adjuvant treatments. In such a case, the employment of sealants can be useful. Following the surgical procedures, the patients were fitted with the Thopaz+ digital drainage system. The purpose of this was two-fold: first, to standardize the management of thoracic drainage (air leakage and fluid collection); and second, to facilitate real-time monitoring of post-operative air leaks. At our institution, following major lung resection, we used two criteria to remove the drain: (1) no air leak during the last 12 h (it means flow 0ml/min on the Thopaz+ device), (2) less than 300 ml of pleural fluid in the last 24h. These criteria remain the same during all the study period.

The post-operative variables collected included: post-operative hemoglobin level, occurrence of PAL (defined as an air leak that persisted for more than five days, as most authors concur on this definition [5, 8, 21, 22]), duration of chest tube drainage, length of hospital stay, 90-day post-operative complications classified as Clavien–Dindo grade>II [23], 90-day readmissions, and 90-day post-operative mortality.

## Methods

### Data collection

We used Epithor database, established in 2002 by the French Society of Thoracic and Cardiovascular Surgery, and recognized through numerous publications [12, 13]. The Ethics Committee for Clinical Research in Thoracic and Cardiovascular Surgery (Registration number IRB00012919) approved the electronic prospective database extraction and use for this study as well as the study itself (Approval number : CERC-SFCTCV-2024-06-25_35090). Patients consented to entry into the prospective database and were aware that these data could be used for clinical research purposes.

### Medical devices

**Fig. 1:**
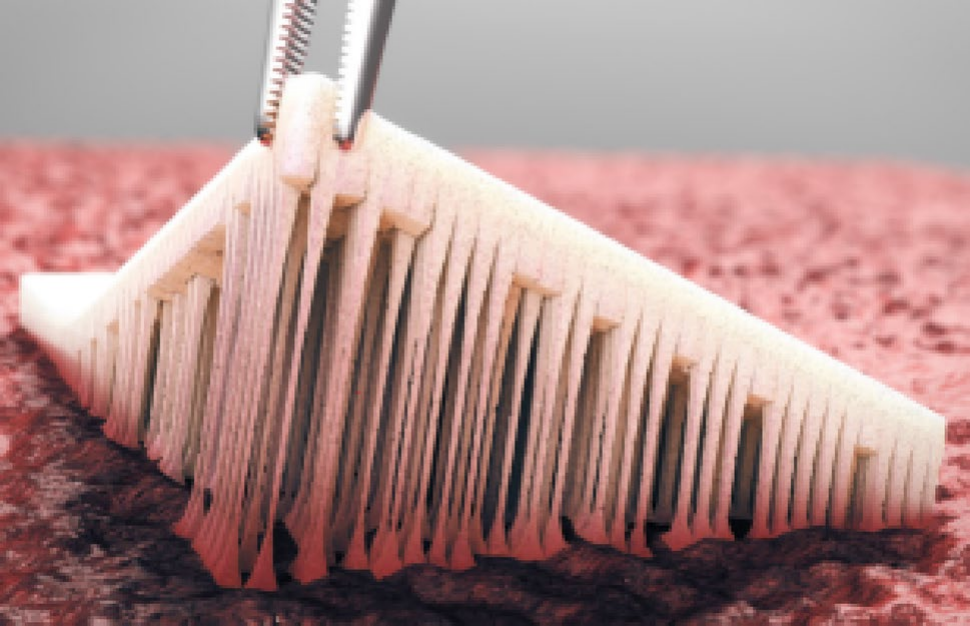
Active surface of TenaTac^®^, covered by more than 1000 columns

The TenaTac^®^ hemostatic patch (patented by Selentus Science, UK and manufactured by CuraMedical, the Netherlands) is a Class III medical device with a European CE Mark, developed for use in surgical procedures. It is manufactured from pharmaceutical-grade gelatine, with no chemical or biological additives. The product’s active side has undergone a physical surface modification laser guided device to create over 1,000 miniscule columns that intersect in a criss-cross pattern. These columns function as a binding mechanism, ensuring that TenaTac^®^ adheres firmly to the surgical site and maintains its position even when the surgeon’s manual pressure is released (Fig. 1). A further distinctive characteristic of TenaTac^®^ is its ability to undergo a gradual transformation in situ into a soft, elastic, translucent adhesive gel upon contact with wet tissues or liquids (e.g., body fluids and saline solution).

In this study, TenaTac^®^ was compared to other devices commonly used by thoracic surgeons for hemostatic and/or aerostatic indications. These products are referred to as hemostatic sealants or glues; for convenience the term sealant is used for all. These sealants in the control group included fibrin glue (1 patient), oxidized regenerated cellulose sealant (5 patients), gelatine-based fibrillary device (10 patients), and polyethylene glycol glue (14 patients).

### Statistical analysis

Statistical analysis was performed using Omni Calculator software. The characteristics of the patients in the two groups and the peri- and post-operative variables were compared. A Student’s t test was utilized to compare the means of the two groups of patients. The means were calculated with consideration for sample size and dispersion (variance). There were no missing data. A significance level of 0.05 was employed for all analyses.

## Results

### Patients’ characteristics and surgical procedures

The patient characteristics are outlined in Table 1. No statistically significant difference was observed between the experimental group (EG) and the control group (CG) with respect to age (*p* = 0.06), gender (*p* = 0.67), BMI (*p* = 0.61), IPAL (*p* = 0.76), ASA score (*p* = 0.76), PS (*p* = 0.80), FEV (*p* = 0.14), dyspnea score (*p* = 0.47), or number of comorbidities (*p* = 0.77).

**Table 1:** Patient characteristics (means and standard deviations)

	Experimental group *N* = 30		Control group *N* = 30		*p* value
Age (years)	62.80 ±15.76		68.91 ±7.75		0.06
Gender	*M* = 53% *n* = 16	*F* = 47% *n* = 14	*M* = 50% *n* = 15	*F* = 50% *n* = 15	0.67
BMI (kg/m^2^)	25.37 ± 5.29		24.72 ± 4.50		0.61
IPAL	7.13 ± 4.86		7.47 ± 3.69		0.76
ASA score	2.38 ± 0.49		2.34 ± 0.55		0.76
Performance status	1.36 ± 0.56		1.40 ± 0.67		0.80
FEV (%)	87.37 ± 14.35		94.21 ± 20.60		0.14
Dyspnea score	1.03 ± 0.72		0.90 ± 0.66		0.47
Number of comorbidities	2.90 ± 2.04		3.03 ± 1.78		0.77
Pre-op Hb level (g/dl)	13.78 ± 1.16		13.62 ± 1.32		0.62
Surgery duration (min)	146.10 ± 27.56		144.00 ± 22.26		0.75

*M* male, *F* female, *IPAL* Index of Prolonged Air Leak, *FEV* forced expired volume in one second, *Pre-op Hb level* pre-operative hemoglobin level

The surgical procedures undertaken in both groups were comparable (28 lobectomies in EG and 29 in CG, 2 segmentectomies in EG and 1 in CG) as well as the localization of the lung resection (13_EG_ *vs* 13_CG_ for RUL; 0 _EG_ *vs* 1 _CG_ for ML; 6 _EG_ *vs* 4 _CG_ for RLL; 7 _EG_ *vs* 7 _CG_ for LUL; 4 _EG_ *vs* 5 _CG_ for LLL) and mean operative duration in both groups (*p* = 0.75). No significant difference was observed in pre-operative hemoglobin levels (*p* = 0.62).

### Hemostatic properties of sealants

The variables displayed no significant difference between EG and CG with regard to intraoperative blood loss (132.21 ± 84.02ml *vs* 144.93 ± 87.37ml, *p* = 0.56) or post-operative hemoglobin levels (12.96 ± 1.51g/dl *vs* 12.64±1.25g/dl, *p* = 0.37). No transfusions were necessary. The TenaTac^®^ patch exhibits equivalent hemostatic properties to sealants commonly used by surgeons in the same circumstances and for the same indications.

### Aerostatic properties of sealants

**Fig. 2:**
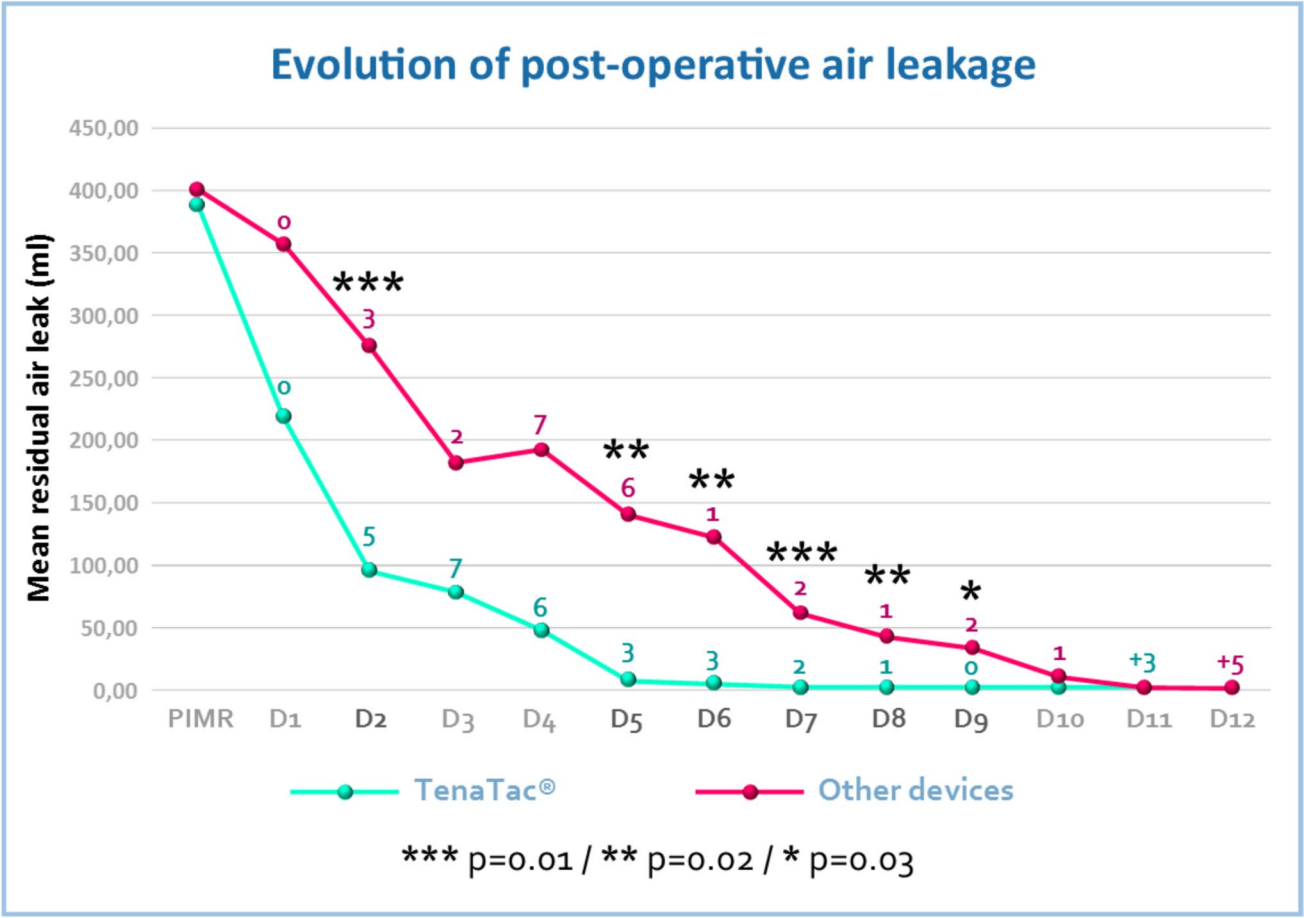
Evolution of post-operative air leakage (significant differences between the two groups of patients are indicated by asterisks). The numerical values indicated above the respective curves correspond to the number of patients from whom the drain was removed. The number of patients who still had the drain after day 10 is indicated at the bottom right of the curve with a “+” mark

No significant differences were observed between the two groups in the immediate post-operative period (data recorded in the recovery room), which indicates that all sealants used are equally effective. However, TenaTac^®^ outperformed the other devices usually used in thoracic surgery from day 2 onwards in terms of residual ongoing air leak volumes (*p* = 0.01). All data collected using the Thopaz+ drainage system (Table 2 and Fig. 2) demonstrate statistically significant differences in favor of TenaTac^®^. The mean times for air leakage (*p* = 0.01), duration of drainage (*p* = 0.04), and hospitalization (*p* = 0.13) were at least 36 hours shorter in the EG than in the CG (Table 3). The PAL rate measured in this study indicates a clear advantage for TenaTac over other sealants: 3% (1 patient) vs 37% (11 patients) with *p* = 0.0004.

**Table 2:** Evolution of post-operative air flows expressed in milliliters/min (means and standard deviations)

	Air flow in experimental group (ml/min)	Air flow in control group (ml/min)	*p* value
Recovery room	389.67 ± 506.04	400.67 ± 482.18	0.47 (NS)
Day 1	220.00 ± 308.71	358.00 ± 485.14	0.10 (NS)
Day 2	95.97 ± 130.24	276.00 ± 399.38	0.01 (**)
Day 3	78.33 ± 206.92	182.67 ± 324.54	0.07 (NS)
Day 4	47.00 ± 167.06	193.00 ± 465.42	0.06 (NS)
Day 5	7.67 ± 24.45	140.33 ± 332.83	0.02 (*)
Day 6	4.33 ± 18.88	122.23 ± 294.84	0.02 (*)
Day 7	2.00 ± 7.61	61.07 ± 139.82	0.01 (**)
Day 8	2.00 ± 7.61	42.07 ± 101.99	0.02 (*)
Day 9	2.67 ± 12.85	33.00 ± 89.14	0.03 (*)
Day 10	1.00 ± 5.48	8.93 ± 33.04	0.10 (NS)
Day 11	0.00	1.67 ± 7.47	0.11 (NS)

*NS* not significant

*p ≤ 0.05, **p ≤ 0.01

**Table 3:** Post-operative follow-up (means and standard deviations)

	Experimental group *N* = 30	Control group *N* = 30	*p* value
Air leak duration (days)	2.23 ± 2.57	4.23 ± 3.87	0.01 (**)
PAL (%)	*n* = 1 3%	*n* = 11 37%	0.0004 (**)
Drainage duration (days)	4.93 ±3.10	6.50 ±3.97	0.04 (*)
Length of stay (days)	7.90 ± 4.66	9.43 ± 5.65	**0.13 (NS)**
Re-draining (%)	*n* = 3 10%	*n* = 5 17%	**0.22 (NS)**
Post-operative complications Clavien–Dindo grade>II (32)	*n* = 6 20%	*n* = 3 10%	**0.47** **(NS)**

*PAL* prolonged air leak, *NS* not significant

*p ≤ 0.05, **p ≤ 0.01

Finally, the incidence of post-operative complications was comparable in both groups. There were no readmissions, re-hospitalizations, reports of serious adverse events, or deaths within three months of surgery (follow-up visit scheduled at 90 days), irrespective of the device utilized.

## Discussion

According to the literature, the prophylactic use of sealants at the site of pulmonary resection appears to be safe and effective. It significantly reduces the intensity and frequency of blood loss and air leaks, the time required for drain removal and, in the majority of cases, the length and cost of hospital stay. These benefits are obtained without compromising clinical outcomes or worsening the post-operative course in terms of morbidity, complications, serious adverse events, or mortality [8].

However, recent surveys and scientific articles have highlighted the need to rationalize their use based on conflicting data related to practitioner skepticism and subjectivity, lack of knowledge about sealants, inherent characteristics and risks of the products employed, high manufacturing costs of some sealants, and even lack of statistically significant benefit compared to conservative treatment [1, 6, 14, 16]. These data do not support the fact that there is systematic use of surgical sealants or tissue reinforcing materials in clinical practice. As evidenced by Rocco et al [24], over 50% of surgeons employed sealing agents when appropriate, while 10% used them routinely. To convince surgeons, the sealant must therefore have maximum therapeutic efficacy based on (1) strong adhesiveness to organic tissues (both to seal the defect and to contribute to wound healing); (2) biocompatibility (to avoid excessive host inflammatory response); (3) controlled degradation (to maintain its functionality during the time required for healing); (4) ad hoc physical properties (in particular flexibility, elasticity and resistance to high pressure) correlated with the applications; and (5) the ability to act in moist conditions [25, 26].

Assmann et al. demonstrated, through in vivo experiments in small and large animal models, that a gelatin-based patch (GelMA) was capable of effectively sealing lung leaks without the need for sutures or staples, while showing improved performance over fibrin glue, polyethylene glycol glue and sutures alone[17].

It is in this context that TenaTac^®^ was introduced. Gelatine, which is analogous to collagen from which it is derived, contains multiple domains that bind to cell surface receptors and extracellular matrix proteins while promoting the attachment and proliferation of mesothelial cells involved in wound healing [27]. The progressive transformation of TenaTac^®^ into a dense gel, characterized by its high level of adhesiveness, elasticity, and flexibility has been demonstrated to effectively control bleeding, presumably via contact activation of the coagulation system and platelet entrapment, and to reduce air leakage via the formation of sealing hydrogel. According to our results, TenaTac^®^ patch exhibits equivalent hemostatic properties to other sealants, it can be applied safely to control hemostasis during major lung resection.

The present outcomes appear to substantiate the observations documented by Grosheva et al [28], in a wide range of surgical specialties. In this study, TenaTac^®^ is described by 40 surgeons from 13 countries as having equivalent or superior efficacy to the sealants they would normally use. The most salient finding of our study is the efficacy of TenaTac^®^ in reducing the incidence of PAL following major lung resection. The prevalence of PAL varies considerably depending on the study, with a commonly accepted average of around 15% to 20% [13, 14, 21, 22]. This complication is a significant challenge for patients and surgeons during the post-operative period as it impedes early discharge and recovery [11]. Consequently, the literature has documented numerous sealants employed over the past four decades to contain air leaks. Some of these products were originally used and well accepted for their hemostatic properties (such as vascular sealants) [7, 9, 15, 22].

Some limitations were present in our study, which included the use of a single site (albeit with three surgeons), retrospective analysis of prospectively collected data, and the use of a sealant at the discretion of the surgeon. Despite the modest sample size, our study demonstrated that TenaTac^®^ led to a substantial reduction in the duration of air leakage and the incidence of PAL. As shown in Fig. 2, a more rapid reduction in air leaks during the initial post-operative period and a superior capacity to prevent PAL were both observed in the TenaTac^®^ group.

Moreover, a mean decrease of 36 hours in drainage and hospitalization times was observed although no statistically significant difference was observed between the two groups with respect to the latter variable. The findings of our study are consistent with those of Takamochi et al [29] on drainage time (estimated at 4.5 ± 2.52 days) and with those of Kent et al [30], assuming that a one-day reduction in drainage time is considered to be of clinical significance. By facilitating the early and efficient removal of the drain, TenaTac^®^ may offer a clinical advantage over other sealants in terms of pain and reduced mobility due to the chest tube.

The issue of operating room utilization time has become a recurrent problem to date. The reduction of operative time is of paramount importance. The swiftness of TenaTac^®^’s utilization and its straightforward management, in conjunction with its storage conditions (ambient temperature) and its immediate availability, serve as compelling arguments for its provision as a promising candidate for substantial enhancement of aerostasis.

While this study provides valuable information on the effectiveness of TenaTac^®^ in perfecting aerostasis, the small sample size and the fact that the study was carried out in a single hospital mean that our results cannot be generalized. As these are the inaugural findings from the application of TenaTac^®^ in thoracic surgery to date, it is imperative to continue investigations on a larger scale, taking into account these preliminary data, in the absence of consensus within the scientific community. The rationale underpinning this proposal is to initiate a multi-center randomized controlled trial with a primary objective on aerostasis.

## Conclusion

TenaTac^®^ seems to be a safe and effective hemostatic and aerostatic patch with performance characteristics that are either equivalent or superior to those of sealants routinely used in thoracic surgery. The application of TenaTac^®^ led to a significant decrease in the duration of air leakage and the rate of PAL at our institution. Given the intrinsic properties of TenaTac^®^, it could be postulated that the patch may improve the immediate post-operative course without compromising patient recovery in terms of post-operative complications. The optimal course of action would be to verify its potential as part of a prospective multi-center randomized controlled trial.

## Data Availability

The authors have full control of all primary data and they agree to allow the journal to review their data if requested.

## Abbreviations

PAL: Prolonged air leak
IPAL: Index of prolonged air leak
ERAS: Enhanced recovery after surgery
EG: Experimental group
CG: Control group
BMI: Body mass index
PS: World health organization performance status
ASA: American society of anesthesiologists
FEV: Forced expiratory volume
RUL: right upper lobe
ML: middle lobe
RLL: right lower lobe
LUL: left upper lobe
LLL: left lower lobe

## Declarations

### Clinical registration information

The study is registered at Health Data Hub (health-data-hub.fr) with the number 1902113.
